# The long-term efficacy of tick-borne encephalitis vaccines available in Europe - a systematic review

**DOI:** 10.1186/s12879-023-08562-9

**Published:** 2023-09-21

**Authors:** Wojciech Miazga, Katarzyna Wnuk, Tomasz Tatara, Jakub Świtalski, Adrian Matera, Urszula Religioni, Mariusz Gujski

**Affiliations:** 1 Department of Health Policy Programs, Department of Health Technology Assessment, Agency for Health Technology Assessment and Tariff System, 00032 Warsaw, Poland; 2 School of Public Health, Centre of Postgraduate Medical Education of Warsaw, Kleczewska 61/63, 01826 Warsaw, Poland; 3 Department of Public Health, Faculty of Health Sciences, Medical University of Warsaw, 02091 Warsaw, Poland; 4 Department of Health Economics and Medical Law, Faculty of Health Sciences, Medical University of Warsaw, 01445 Warsaw, Poland

**Keywords:** Encephalitis, Tick-borne, Vaccines, Immunization, TBE

## Abstract

**Background:**

Despite the availability of vaccination, TBE (tick-borne encephalitis) remains a global public health problem. Therefore, the aim of our study was to assess the long-term efficacy of vaccinations against tick-borne encephalitis using vaccines available on the European market.

**Methods:**

The analysis was conducted on the results of a systematic review conducted in accordance with the Cochrane Handbook for Systematic Reviews of Interventions. The search was performed in three databases, namely Medline (via PubMed), EMBASE (via Ovid), and the Cochrane Library database. The authors followed the PRISMA method and the selection of the articles was performed with two independent researchers.

**Results:**

From a total of 199 citations, 9 studies were included in this review. According to the primary studies identified in the search, the efficacy of available anti-TBE vaccines ranges from 90.1% to 98.9%; however, in individuals above the age of 60, the protection wanes as early as one year after vaccination. Administration of a booster dose 3 years after completion of the basic vaccination schedule significantly extended the period of protection against TBE.

**Conclusions:**

Anti-TBE vaccines available in Europe have a high level of efficacy. However, the level of protection against TBE is decreasing after vaccination. Therefore, in addition to the conventional schedule, booster vaccines should be administered every 5 years in individuals before the age of 60 and more frequently, e.g. every 3 years, in individuals aged 60 and beyond.

**Supplementary Information:**

The online version contains supplementary material available at 10.1186/s12879-023-08562-9.

## Background

Tick-borne encephalitis (TBE) is a zoonotic disease with clinical presentation corresponding to that of aseptic meningitis and/or encephalitis. The disease is endemic in Central Europe, the Baltic region, Russia, and parts of eastern Asia. The etiological pathogen responsible for the disease is the Central European Encephalitis virus of the *Flaviviridae* family, transmitted mainly by infected ticks and present in non-pasteurized dairy products derived from animals living within endemic areas. Tick-borne encephalitis viruses (TBEV) are divided into main three subtypes: European (TBEV-EU), Siberian (TBEV-SIB), and far Eastern (TBEV-FE). However, there are publications that mention the division into more subtypes, e.g.: TBEV-Ob (TBEV-2871), TBEV-Him, TBEV-Bkl-1 (178–79) i TBEV-Bkl-2 (886–84) [1–3]. The disease requires laboratory confirmation due to the non-specific symptoms which can be also found in other forms of meningitis/encephalitis [4]. However, it is worth noting that most of patients infected with TBEV are asymptomatic. According to data from the European Surveillance System (TESSy), which collects analyses and publishes data on infectious diseases, a total of 3,817 cases of tick-borne encephalitis were reported in 2020 in 24 EU/EEA countries. The greatest numbers of cases were recorded in Czech Republic, Lithuania, and Germany. Cases were most commonly reported in male subjects within the age group of 45–64 [5].

There is no specific treatment method for TBE. Suspect patients are treated symptomatically, including administration of antipyretic, analgesic, antiemetic, and antiedematous agents [6].

Two preventive strategies are used to protect populations against the disease, namely actions aimed at prevention against tick bites and vaccinations against the virus causing the disease.

In order to prevent tick bites, the International Association for Medical Assistance to Travelers recommends that individuals undertaking outdoor activities in forest areas in endemic regions:

- use repellent products containing 20–30% of DEET or 20% of picaridin;
- wear long-sleeved shirts and long trousers with trouser legs slipped under socks;
- where possible, avoid high grasses and shrubs.

In addition, the organization recommends careful examination of one’s body, clothing, equipment, and pets for any attached ticks prior to returning home [7].

In Europe, two inactivated vaccines against TBE, developed from cell cultures, are available in adult and pediatric forms: FSME-IMMUN (TicoVac) (manufacturer: Pfizer) and Encepur (manufacturer: Bavarian Nordic). In Russia, two inactivated vaccines against TBE are available: Kleshch-E-Vak (manufacturer: Chumakov Institute of Poliomyelitis and Viral Encephalitis) and EnceVir (manufacturer: Microgen). China produces the inactivated vaccine under the name of SenTaiBao (manufacturer: Changchun Institute of Biological Products in China) [8]. In 2021, the FDA approved TicoVac (manufacturer: Pfizer) for use in the US by individuals intending to travel to areas of endemic prevalence of TBE [9].

## Methods

- The systematic review was conducted in accordance with the Cochrane Handbook for Systematic Reviews of Interventions [10], including the following:definition of the criteria for the inclusion of studies in the review;
- development/verification of the strategy of the search for scientific reports;
- querying/requerying medical information sources;
- identification of full-text reports potentially useful in clinical analysis;
- selection of studies based on inclusion criteria;
- processing of study results;
- qualitative synthesis consisting in the analysis of the statistical and clinical significance of the results of studies included in the analysis.

The search for clinical trials was based on a detailed protocol defined prior to the start of the study. The inclusion criteria, search strategy, selection algorithm and the planned analytical methodology were taken into account.

Included in the analysis were clinical trials meeting the predefined criteria regarding:

- population: general population;
- intervention: completion of the full conventional basic anti-TBE vaccination schedule;
- alternative technologies (comparators): not limited;
- methods: experimental studies (randomized clinical trials, non-randomized clinical trials with pre-test/post-test control group design, single-arm trials, post-registration studies), and observational controlled studies (case–control, ecological, and cohort studies).
- endpoints: vaccination efficacy, seropositivity (development of immunity against TBE as measured by neutralization test results NT ≥ 10), geometric mean titer (GMT) of antibodies.

The search for primary studies was carried out in the following sources of medical information: the Medline (via PubMed), the EMBASE (via Ovid), and the Cochrane Library databases. The last database query was performed on November 25, 2022. The main Mesh terms used in the search strategy included: encephalitis, tick-borne; vaccines; immunity. The search strategies available in supplementary materials.

At all stages of the systematic review, the selection of research studies was performed independently by two analysts (K.W. and W.M.). Any disagreements were resolved by consensus with the participation of a third independent analyst (A.M.). The causes for the exclusion of studies from the analysis were related to:

- the intervention used in the publication did not meet the inclusion criterion for the review (*n* = 4),
- the population described in the publication did not meet the inclusion criteria for the review (*n* = 1),
- research methodology raised objections or did not describe the indicators analyzed in the review (*n* = 4),
- access to the full text of the publication was not obtained (n = 4). The list of publications included in the review and excluded from the review can be found in the Supplementary Materials.

The quality of non-randomized trials included in the review was assessed using the ROBINS-1 tool [11]. The only randomized control trial (RCT) included in the review was assessed using the risk-of-bias tool (RoB 2 tool) [12]. The quality assessments were carried out independently by two analysts (A.M. and K.W.). Any disagreements were resolved by consensus.

## Results

**Fig. 1:**
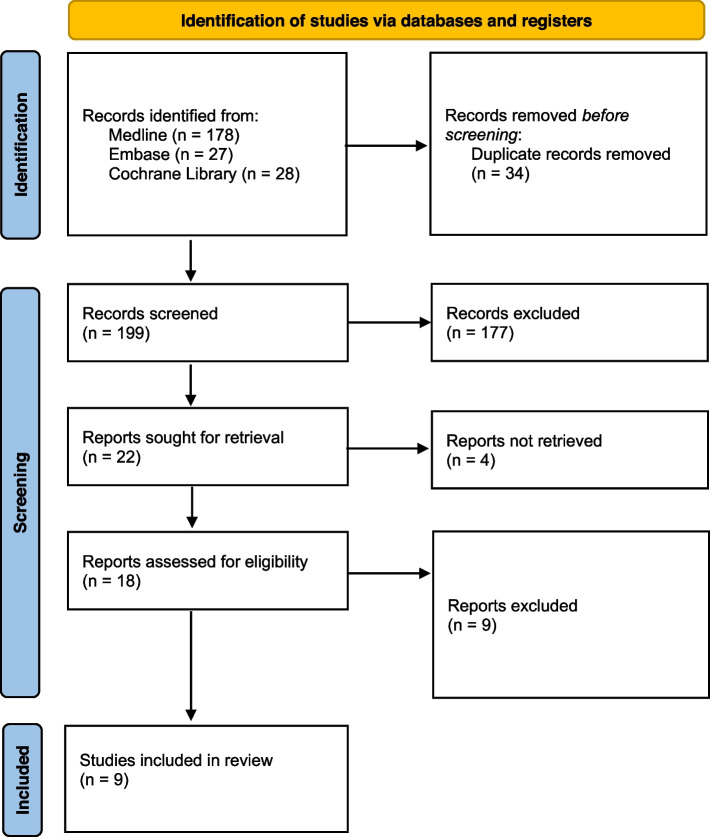
PRISMA flow diagram

Nine studies met the inclusion criteria and were included in the review. The types of studies included were ecological study (*n* = 1) [13], randomized open clinical trial (*n* = 2) [14, 15], single-arm studies (*n* = 4) [16–19], case control study (*n* = 2) [20, 21], The steps for the selection of studies were shown in Fig. 1. The authors followed the PRISMA method.

**Fig. 2:**
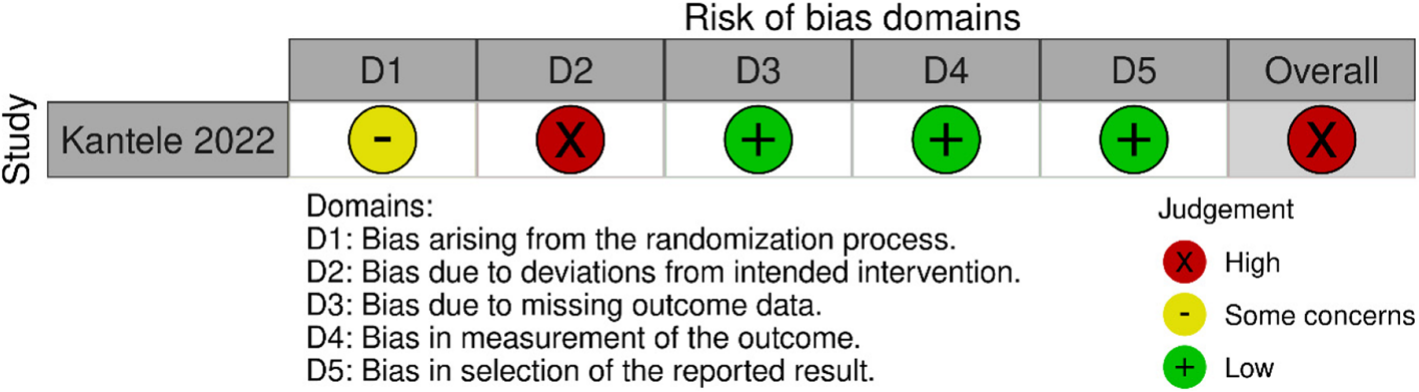
Quality assessments (RoB 2)

**Fig. 3:**
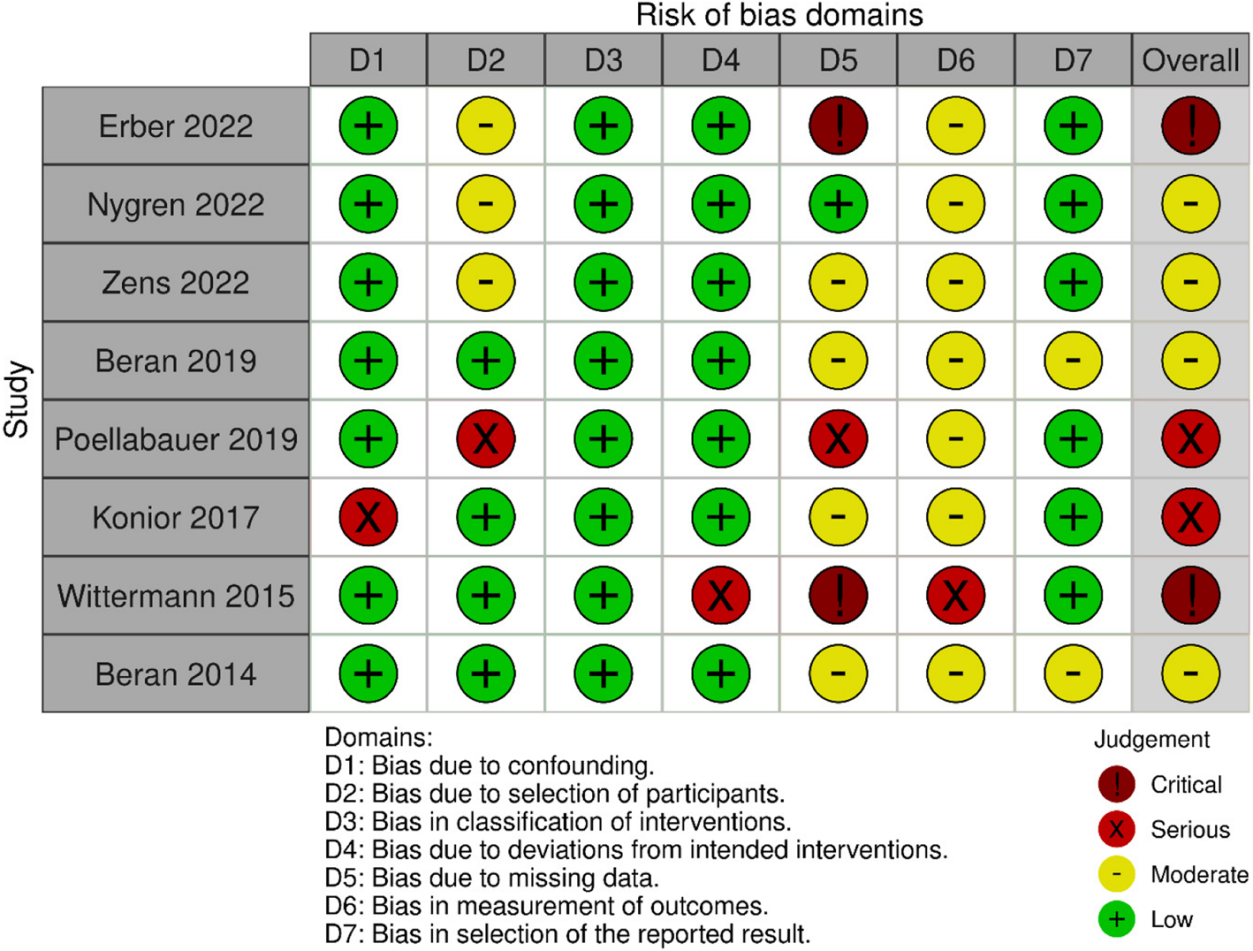
Quality assessments (ROBINS-I)

None of the studies included in the analysis was characterized by a low risk of systematic error; the predominant risk level was moderate (*n* = 4). The identified studies were sufficient to conclude on the effectiveness of anti-TBE vaccinations. Detailed results of the quality and risk of bias analyses are shown in Figs. 2 and 3.

The results of the identified studies are presented below.

### The efficacy of anti-TBE vaccinations

The Erber 2022 ecological study assessed the efficacy of preventive anti-TBE vaccinations in the residents of highly endemic areas in Latvia and Germany. In this case, the efficacy of vaccination was defined as the percentage of subjects who did not develop TBE following vaccination with FSME-Immun or Encepur vaccines. In the case of endemic areas in Germany (Bavaria and Baden-Württemberg), completion of the full 3-dose vaccination schedule and subsequent administration of the booster dose had the overall efficacy of 92.8% [95%CI: (0.909–0.943)] and 95.4% [95%CI: (0.939–0.965)], respectively. For the endemic areas in Latvia, the efficacy of the full (3-dose) vaccination schedule was 98.9% [95%CI: (0.981–0.994)]. Following the administration of the booster dose, the vaccination efficacy was 98.8% [95%CI: (0.982–0.993)]. The authors indicated that the time since the last dose of the vaccine had no negative effect on VE [13].

The Nygren 2022 case–control study assessed the efficacy of preventive vaccinations within the TBE-endemic areas in Germany. The efficacy of vaccination was measured using a conditional logistic regression model calculated using the formula VE (vaccine effectiveness) = [1 − OR] × 100. The reported efficacy of anti-TBE vaccination was 96.6% [95%CI: (0.937–0.982)] for a 3-or-more-dose regimen administered as recommended by the manufacturer, including the recommended intervals. With respect to the product used, the Encepur and FSME-Immun vaccines had the efficacy of 95.8% [95%CI: (0.897–0.983)] and 90.1% [95%CI: (0.866–0.965)], respectively. If one booster dose was administered after completion of the basic schedule, the vaccine efficacy increased to 96.6% [95%CI: (0.937–0.982)]. Completion of the basic 3-dose regimen translated to the efficacy of 91.9% [95%CI: (0.852–0.956)]. However, when the intervals were exceeded > 3 or > 5 years but the last vaccine dose was within 10 years, the VE was 91.2%. When the last dose was > 10 years ago, the VE was 88.6%. These results were stable across all age groups [20].

The Zens 2022 case–control study assessed the efficacy of 3 or more doses of anti-TBE vaccines administered as part of immunization regimens in Switzerland. The analysis was performed with the time since the last dose of vaccination (3 or more doses) being taken into account as a parameter. Similarly to the Nygren 2022 study, vaccination efficacy was calculated as VE = [1 − OR] × 100. When the last dose had been administered 5 years prior to the study, the vaccination efficacy was at the level of 91.6% [95%CI: (0.884–0.940)]. When the last dose had been administered 5–10 years prior to the study, the vaccination efficacy reached the level of 95.2% [95%CI: (0.924–0.970)]. In the group of individuals who had had their last dose of vaccine administered more than 10 years prior to the study, the efficacy was at the level of 98.5% [95%CI: (0.968–0.992)]. The overall efficacy in the general population amounted to 95.0% [95%CI: (0.935–0.961)] [21].

The characteristics and results of the anti-TBE vaccine efficacy studies are presented below (Table 1).

**Table 1:** Characteristics and results of the anti-TBE vaccine efficacy studies

Author/year	Type of study	Follow-up period	Vaccination	Population			Vaccination schedule		VE (%) (95%CI)
				**Population**		Population size (n/N)*			
Erber 2022 [13]	Ecological study	12 years 2007–2018	FSME-Immun®/Encepur®	Residents of areas of endemic prevalence	Germany (Bavaria and Baden-Württemberg)	75/3,366,349 (I) 3,010/9,747,843 (C)	Completion of the basic schedule (3 doses)		92.8 (0.909–0.943)
						50/3,530,490 (I) 3,010/9,747,843 (C)	Administration of the first booster dose after completing the basic schedule		95 (0.939–0.965)
					Latvia	13/308.420 (I) 3,044/800,481 (C)	Completion of the basic schedule (3 doses)		98.9 (0.981–0.994)
						18/407.183 (I) 3,044/800,481 (C)	Administration of the first booster dose after completing the basic schedule		98.8 (0.982–0.993)
Nygren 2022 [20]	A case control study	–2020	FSME-Immun®/Encepur®	Residents of the areas of endemic prevalence in Bavaria and Baden-Württemberg (Germany)		13/235	Completion of the full vaccination schedule (≥ 3 doses) administered on time as recommended by the manufacturer		96.6 (0.937–0.982)
						8/105	Completion of the full ENCEPUR vaccination schedule (≥ 3 doses)		95.8 (0.897–0.983)
						13/106	Completion of the full FSME-IMMUN vaccination schedule (≥ 3 doses)		90.1 (0.866–0.965)
						16/145	Completion of the basic schedule (3 doses)		91.9** **(0.852–0.956)
						20/280	Administration of the first booster dose after completing the basic schedule (4 doses)		95.9** (0.926–0.977)
Zens 2022 [21]	A case control study	5, 5–10, and 10 years	FSME-Immun®/Encepur®	Individuals aged 18 to 79, with or without TBE, residing in Switzerland in years 2006–2020		48/522	Completed vaccination schedule (3 or more doses)	< 5 years	91.6 (0.884–0.940)
						20/397		5–10 years	95.2 (0.924–0.970)
						8/458		> 10 years	98.5 (0.968–0.992)
						76/1377		Total	95 (0.935–0.961)

(I) investigation group, (C) control group, VE – vaccine effectiveness

^*^ *N* case, *N* number of people in the intervention or control group

^**^ univariable estimates

### Seroprevalence and persistence of anti-TBE antibodies

#### Basic schedule

The Kantele 2022 RCT addressed the question of achieving seropositivity and geometric mean titer (GMT) of antibodies following anti-TBE vaccination with FSME-Immun 0.5 mL according to the standard 3-dose regimen. The follow-up period covered a total of 400 days after vaccination. In the population of subjects aged < 50 years, NT ≥ 10 (seropositivity) was found in 96% (0.86–1) of subjects, with GMT at the level of 74 [95%CI: (49–111)]. Both the percentage of seropositive individuals and the GMT value were slowly decreasing with age. This could be observed in the population of subjects aged 50–59, where the seropositivity rate was 96% [95%CI: (0.79–1)] and GMT was at the level of 65 [95%CI: (36–117)], as well as in the population of subjects aged ≥ 60, where the seropositivity rate was 74% [95%CI: (0.52–0.90)] and GMT was at the level of 26 [95%CI: (12–55)] [14].

In the Wittermann 2015 phase IV post-registration study, the authors addressed the issue of seropositivity and GMT in the population of children aged 5–15 years. The follow-up timepoints were defined at 3, 4, and 5 years after Encepur Children vaccine was administered in the basic immunization regimen. With regard to the seropositivity rate, the value of 100% [95%CI: (0.93–1)] was maintained up to the 4^th^ year of observation. In the fifth year, a slight decrease to the level of 98% [95%CI: (0.89–1)] was observed. With regard to the GMT, its value after 3 years of follow-up amounted to 497 [95%CI: (302–819)] and increased to 601 [95%CI: (424–854)] after one year while dropping to 337 [95%CI: (228–498)] 5 years after vaccination [15].

#### Booster doses

The Beran 2019/2014 single-arm studies determined the proportion of seropositive patients and GMT values in subjects aged 15–60 years and having received a booster dose of Encepur Adults following the completion of the basic immunization schedule. The follow-up timepoints were defined at 5, 6, and 10 years. With regard to the seropositivity rate, the value of 100% [95%CI: (0.93–1)] was maintained through the 6^th^ year of observation. In the tenth year, a slight decrease to the level of 98% [95%CI: (0.89–1)] was observed. With regard to GMT, its value after 5 years of follow-up amounted to 300 [95%CI: (196–460)] and decreased to 293 [95%CI: (200–428)] after one year while reaching 307 [95%CI: (202–466)] in the fifth year [16, 17].

The single-arm Poellabauer 2019 study presents the changes in GMT values and seropositivity rates over a period of 118 months after booster vaccination with FSME-IMMUN® at a 0.25-mL or a 0.5-mL dose. With regard to the seropositivity rate, the value of 100% was maintained over the first 46 months. In the following months, it began to gradually decrease by about 1–2%, reaching 90.3% [95%CI: (0.845–0.945)] at the final follow-up timepoint. The GMT value in the first follow-up period was 380.7 [95%CI: (336.73–430.31)]. Over time, the value gradually decreased and reached 53.9 [95%CI: (43.40–66.87)] on the last day of the follow-up period [18].

The results of the single-arm Konior 2017 study support those of the Poellabauer 2019 study. The follow-up periods and the target population remained unchanged, the only difference in the methodology consisting in FSME-IMMUN® being used only at the dose of 0.5 mL. With regard to the seropositivity rate, the value of 100% was maintained in the initial period [95%CI: (0.985–1)]. Subsequently, the value began to decrease to reach 84.9% [95%CI: (0.803–0.887)] at 118 months. The GMT value in the first follow-up period was 450.4 [95%CI: (421.72–480.93)]. In subsequent periods, its value gradually decreased in a manner similar to that observed in the Poellabauer 2019 study, reaching a level of 37.0 [95%CI: (29.12–47.05)] at 118 months of follow-up [19].

The characteristics and individual results of the studies on seropositivity rates and persistence of antibodies (GMT) following anti-TBE vaccinations are presented in the Table 2.

**Table 2:** Characteristics and results of the studies on seropositivity rates and geometric mean titers of antibodies (GMT) following anti-TBE vaccinations

Author/year	Type of study	Vaccination	Population		Follow-up time point	NT ≥ 10 (%)^a^	GMT^b^
			**Description**	**N**		**(95%CI)**	
**Basic schedule**							
Kantele 2022 [14]	Single-center, open-label, randomized controlled trial	FSME-IMMUN® 0.25 mL (0, 30, 360)	Healthy subjects aged ≥ 50 years (intervention group) and < 50 years (control group)	50 (< 50 years)	400 days	96 (0.86–1)	74 (49–111)
				49 (50–59 years)		96 (0.79–1)	65 (36–117)
				46 (≥ 60 years)		74 (0.52–0.90)	26 (12–55)
Wittermann 2015 [15]	Phase IV post-registration study	Encepur® Children (0, 28, 300)	Healthy children and adolescents aged 5–15 years, previously included in the primary clinical trial comparing different anti-TBE vaccination schedules	50	3 years	100 (0.93–1)	497 (302–819)
					4 years	100 (0.93–1)	601 (424–854)
					5 years	98 (0.89–1)	337 (228–498)
**Administration of a booster dose after completion of the basic conventional schedule**							
Beran 2019/2014 [16, 17]	A single-arm phase IV study (per protocol population)	Encepur Adults®	Individuals aged 15 to 60 who had received a booster dose 3 years after completing the basic vaccination schedule (0, 28, 300)	48	5 years	100 (0.93–1)	300 (196–460)
				51	6 years	100 (0.93–1)	293 (200–428)
				49	10 years	98 (0.89–1)	307 (202–466)
Poellabauer 2019 [18]	A single-arm study	FSME-IMMUN® 0.25 mL (< 16 years) FSME-IMMUN® 0.5 mL (< 16 years)	Individuals having received the complete basic vaccination schedule and eligible for another booster dose	171	21–35 days	100 (0.98–1)	380.7 (336.73–430.31)
				167	38 months	100 (0.98–1)	162.1 (139.29–188.58)
				147	46 months	100 (0.98–1)	108.1 (92.41–126.34)
				156	58 months	99.4 (0.97–1)	111.3 (96.10–128.84)
				157	70 months	98.1 (0.95–0.99)	123.4 (105.31–144.59)
				156	82 months	96.8 (0.93–0.98)	122.3 (102.33–146.28)
				156	94 months	95.5 (0.91–0.98)	82.8 (68.88–99.57)
				156	106 months	94.9 (0.90–0.98)	56.0 (46.42–67.57)
				156	118 months	90.3 (0.85–0.95)	53.9 (43.40–66.87)
Konior 2017 [19]	A single-arm study	FSME-IMMUN® 0.5 mL	Adults having received the complete basic vaccination schedule and eligible for another booster dose	315	21–35 days	100 (0.99–1)	450.4 (421.72–480.93)
				314	27 months	99.7 (0.98–1)	108.7 (97.22–121.47)
				405	34 months	100 (0.99–1)	129.0 (115.79–143.68)
				305	46 months	96.1 (0.93–0.98)	119.0 (105.35–134.51)
				304	58 months	95.4 (0.92–0.98)	97.7 (85.07–112.27)
				304	82 months	89.8 (0.86–0.93)	78.6 (65.27–94.67)
				304	94 months	88.2 (0.84–0.92)	93.6 (75.16–116.60)
				304	106 months	86.2 (0.82–0.89)	58.2 (46.20–73.34)
				304	118 months	84.9 (0.80–0.89)	37.0 (29.12–47.05)

*CI* confidence interval, *N* number of people

^a^Seroprevalence (neutralization test: NT ≥ 10) defined as the percentage of subjects with NT ≥ 10

^b^GMT – geometric mean titer

## Discussion

### Summary of evidence

In 2020, a systematic review on the immunogenicity and safety of tick-borne encephalitis vaccinations was published; included in the review were publications from years 2009–2019 [22]. The review showed that the vaccines were safe and their efficacy was high, albeit waning in subjects older than 50 years. It was therefore concluded that the age at which the vaccination schedule is started is the key factor affecting the length of protection. After 2019, further scientific reports were published on the efficacy of vaccination and the persistence of antibodies against TBE following vaccination, which was the reason for the undertaking of this systematic review of primary studies.

Based on the results of the studies identified in the systematic search, the efficacy of anti-TBE vaccination (e.g. defined as the percentage of subjects who did not develop TBE following vaccination) was assessed and the issues of prevention and persistence of anti-TBE antibodies were addressed.

Taking into account the results of the studies on the efficacy of vaccination, it is possible to conclude that the efficacy of vaccinations in the prevention of TBE is high and ranges from 90.1% to 98.9% [13, 20, 21].

In the Kantele 2022 study based on a 3-dose FSME-IMMUN® schedule (days 0, 30, 360), the GMT value 400 days after vaccination in the group of individuals under the age of 50 was at the level of 74 [95%CI: (49–111)]. The analysis of the publication data shows that GMT decreased with age, with a significant decrease being observed in the group of subjects above 60 years of age. (NT ≥ 10: 74%, GMT: 26) [14]. In the Wittermann 2015 post-registration phase IV study carried out in a population of subjects aged 5–15 years (Encepur® Children on days 0, 28, 300), high GMT levels were demonstrated, although significant differences and a gradual decrease were observed over the consecutive years. Three years after vaccination, the GMT was at the level of 497 [95%CI: (302–819)] while dropping to 337 [95%CI: (228–498)] after 5 years [15]. In view of the above data, the administration of vaccine boosters is worth considering every 5 years in individuals before the age of 60 and more frequently, in individuals aged 60 and beyond. The results of the studies on booster doses administered after completion of basic immunization schedules suggest a gradual decrease in the level of seroprevalence after 58 months [18] or 46 months of follow-up [19], depending on the publication.

In addition to the systematic screening for scientific evidence, the search pertained to current management guidelines and recommendations in different countries of the world. Vaccination against TBE is recommended by scientific organizations as well as by governmental and non-governmental healthcare entities all over the world as one of the methods for the prevention of the disease in individuals staying or working in areas where ticks are common as well as in individuals traveling to areas of endemic prevalence of TBE [23–33]. Professions at the highest risk of exposure to ticks include foresters, farmers, military personnel, or researchers performing field work [24, 25, 31]. In some recommendations, examples of outdoor activities involving the risk of TBE infections are also listed, and include hiking, camping, running, cycling, hunting, fishing, bird watching, and picking mushrooms, flowers, or berries [23, 24, 26]. Some of the identified papers suggest the plausibility of vaccinations against TBE being performed in all individuals above 1 year of age living in areas with an endemic prevalence of the disease, with the annual incidence rate at the level of ≥ 5/100,000 [25, 30, 33].

### Immunization schedules

The European Centre for Disease Prevention and Control (ECDC) platform collects information on preventive immunization schedules within the countries of the European Union and the European Economic Area. According to information available from the ECDC platform, anti-TBE vaccinations are reimbursed in two countries [34]. In Latvia, vaccines are reimbursed for residents of areas of endemic prevalence starting from the age of 1 year [34, 35]. In Slovenia, vaccinations are carried out in children aged 3 years (3 vaccine doses, additional doses self-paid) and adults aged 49 years (3 vaccine doses, additional doses self-paid) [34]. In several countries vaccination is recommended but not reimbursed from public funds. For example, this is the case in Austria where the basic schedule of 3 doses is recommended, with the first booster dose to be administered after 3 years and the subsequent boosters to be administered every 5 years until the age of 60, and every 3 years thereafter. In the Czech Republic, vaccination with a 3-dose schedule is recommended along with booster to be administered every 5 years (for individuals aged 1–59 years) or every 3 years (individuals aged 60 or above). In Finland, vaccination is recommended for all individuals aged 3 and over who are permanent residents of the island of Åland [34]. In Poland, preventive vaccination against TBE is recommended for people living in areas with an increased prevalence of this disease, in particular for people employed in forestry, military personnel, fire and border guards, farmers, young people on internships and professional practices, as well as individuals who frequently engage in outdoor activities (e.g. joggers, hikers, mushroom pickers, dog owners, hunters, etc.) as well as other individuals undertaking outdoor activities, in particular tourists and camp participants [36]. In other countries (e.g. Switzerland, Sweden, Germany, Israel, Kazakhstan), vaccinations against TBE are also recommended for specific groups of individuals [22, 37].

### Implication for research and health policies

Increasingly, there is a discussion about the effectiveness of vaccinations in the long term and the time interval to receiving subsequent booster doses [38]. There are studies showing that delayed timing after taking the 4th dose did not significantly affect the effectiveness of vaccination in any of the analyzed age groups [13]. In another study, after taking the 4th dose seroprevalence after 10 years of observation, was still at a very high level [17]. Further studies are needed to evaluate the effectiveness of vaccination schedules over an even longer period of follow-up. Studies evaluating the impact of successive doses of the vaccine and their intervals on the effectiveness of prophylaxis would also be useful. This is an important issue that can affect your willingness to vaccinate and adhere to vaccination schedules. Vaccination attendance is currently very diverse in European countries [39], and one of the factors that may influence reluctance to be vaccinated is the need to take booster doses.

For these reasons, work should also be carried out on the creation of novel vaccination strategies, while taking into account and thoroughly understanding the immune correlates of protection against TBEV. However, these issues also require further research [40].

### Limitations of the review

English-language publications only were included in the review. The search was restricted to studies published in the last 10 years (25 November 2012 – 25 November 2022). The studies found were characterized by high heterogeneity and diverse methods of data presentation. In addition, it should be borne in mind that the identified publications originate from different countries with different risks of acquiring TBE.

## Conclusions

Tick-borne encephalitis is a disease which, although not common on a global scale, is nonetheless very dangerous as it leads to serious neurological complications; in extreme cases, it may also lead to death. For this reason, the prevention of TBE is very important. Vaccination is an effective method for prevention of the disease.

The results of the studies identified in the search are suggestive of a very high efficacy of vaccinations. However, one should bear in mind that the level of protection is steadily decreasing after vaccination. A significant decrease in protection has also been observed with age. Therefore, following basic vaccination according to the conventional schedule, booster vaccines should be administered every 5 years in individuals before the age of 60 and more frequently, e.g. every 3 years, in individuals aged 60 and beyond.

However, evidence is emerging to suggest that booster intervals may be longer andthis requires further, well-designed research.

Vaccinations should be routinely performed in the areas of endemic prevalence of TBE with annual incidence rates of ≥ 5/100,000. People from risk groups (e.g. foresters, farmers, military personnel) who live in a low endemic area with emerging cases should also be vaccinated. Individuals traveling to the areas of the endemic prevalence of TBE should also be vaccinated before arrival.

### Supplementary Information

**Additional file 1:** **Table S1.** Search strategy Cochrane. **Table S2.** Search strategy Medline (via PubMed). **Table S3.** Search strategy Embase (via Ovid). **Table S4.** List of studies included and excluded after full-text analysis.

## Data Availability

All data are available from the corresponding author.

## Abbreviations

DEET: N,N-Diethyl-meta-toluamide
GMT: geometric mean titer
RCT: Randomized controlled trial
RoB: risk-of-bias
TBE: Tick-borne encephalitis
TBEV: Tick-borne encephalitis viruses
TESSy: the European Surveillance System
VE: vaccine effectiveness
