# Supplementary material for: The long-term efficacy of tick-borne encephalitis vaccines available in Europe - a systematic review
Source: BMC Infect Dis. 2023 Sep 21;23:621. doi: 10.1186/s12879-023-08562-9 (PMC10515056; doi:10.1186/s12879-023-08562-9)
Supplement: Supplementary file 1 — Additional file 1: Table S1. Search strategy Cochrane. Table S2. Search strategy Medline (via PubMed). Table S3. Search strategy Embase (via Ovid). Table S4. List of studies included and excluded after full-text analysis. [file 12879_2023_8562_MOESM1_ESM.docx]

**Table S1. Search strategy Cochrane**

| ID | Keyword | Result |
| --- | --- | --- |
| #1 | MeSH descriptor: [Encephalitis, Tick-Borne] explode all trees | 53 |
| #2 | (Tick-Borne Encephalitis):ti,ab,kw | 92 |
| #3 | (TBE):ti,ab,kw | 96 |
| #4 | #1 OR #2 OR #3 | 129 |
| #5 | MeSH descriptor: [Vaccines] in all MeSH products | 14221 |
| #6 | (vaccin*):ti,ab,kw | 29605 |
| #7 | MeSH descriptor: [Immunity] in all MeSH products | 4198 |
| #8 | (immun*):ti,ab,kw | 139617 |
| #9 | #5 OR #6 OR #7 OR #8 | 147112 |
| #10 | #4 AND #9 with Publication Year from 2012 to 2022, in Trials | 28 |

**Table S2. Search strategy Medline (via PubMed)**

| ID | Keyword | Result |
| --- | --- | --- |
| #13 | Search: #10 AND #11 Filters: from 2012 - 2022 | 178 |
| #12 | Search: #10 AND #11 | 415 |
| #11 | Search: ((((((((((study* OR trial* OR trail* OR experiment*[Title/Abstract]))) AND ((control OR random* OR blind* OR mask*[Title/Abstract])))) OR (("Randomized Controlled Trial" [Publication Type] OR "Randomized Controlled Trials as Topic"[Mesh] OR "Controlled Clinical Trial"))))))) | 4001271 |
| #10 | Search: #4 AND #9 | 2300 |
| #9 | Search: #5 OR #6 OR #7 OR #8 | 2980950 |
| #8 | Search: immun*[Title/Abstract] | 2728664 |
| #7 | Search: Immunization[MeSH Terms] | 203114 |
| #6 | Search: vaccin*[Title/Abstract] | 396295 |
| #5 | Search: vaccine[MeSH Terms] | 268154 |
| #4 | Search: #1 OR #2 OR #3 | 6152 |
| #3 | Search: TBE[Title/Abstract] | 2347 |
| #2 | Search: Tick-Borne Encephalitis[Title/Abstract] | 4665 |
| #1 | Search: Tick-Borne Encephalitis[MeSH Terms] | 3496 |

**Table S3. Search strategy Embase (via Ovid)**

| ID | Keyword | Result |
| --- | --- | --- |
| 1 | exp Tick-Borne Encephalitis/ | 3148 |
| 2 | Tick-Borne Encephalitis.ab,kw,ti. | 3818 |
| 3 | TBE.ab,kw,ti. | 2632 |
| 4 | 1 or 2 or 3 | 5737 |
| 5 | exp vaccine/ | 349513 |
| 6 | "vaccin*".ab,kw,ti. | 421864 |
| 7 | "vaccin*".ab,kw,ti. | 421864 |
| 8 | "vaccin*".ab,kw,ti. | 421864 |
| 9 | 5 or 6 or 7 or 8 | 3563567 |
| 10 | 4 and 9 | 2390 |
| 11 | limit 10 to ((clinical trial or randomized controlled trial or controlled clinical trial) and yr="2012 -Current") | 27 |

**Table S4. List of studies included and excluded after full-text analysis**

| **Lp.** | **Authors, Title, Journal** | **Full text status** | **Reason for exclusion** |
| --- | --- | --- | --- |
| 1. | Aerssens A, Cochez C, Niedrig M, Heyman P, Kühlmann-Rabens I, Soentjens P. Analysis of delayed TBE-vaccine booster after primary vaccination. J Travel Med. 2016 Feb 8;23(2):tav020. doi: 10.1093/jtm/tav020. PMID: 26858269. | Excluded | Intervention |
| 2. | Askling HH, Vene S, Rombo L, Lindquist L. Immunogenicity of delayed TBE-vaccine booster. Vaccine. 2012 Jan 11;30(3):499-502. doi: 10.1016/j.vaccine.2011.11.061. Epub 2011 Nov 26. PMID: 22122857. | Excluded | Intervention |
| 3. | **Beran J, Lattanzi M, Xie F, Moraschini L, Galgani I. Second five-year follow-up after a booster vaccination against tick-borne encephalitis following different primary vaccination schedules demonstrates at least 10 years antibody persistence. Vaccine. 2019 Jul 26;37(32):4623-4629. doi: 10.1016/j.vaccine.2017.12.081. Epub 2018 Feb 1. PMID: 29397225.** | **Included** | **-** |
| 4. | **Beran J, Xie F, Zent O. Five year follow-up after a first booster vaccination against tick-borne encephalitis following different primary vaccination schedules demonstrates long-term antibody persistence and safety. Vaccine. 2014 Jul 23;32(34):4275-80. doi: 10.1016/j.vaccine.2014.06.028. Epub 2014 Jun 17. PMID: 24950352.** | **Included** | **-** |
| 5. | Costantini M, Callegaro A, Beran J, Berlaimont V, Galgani I. Predicted long-term antibody persistence for a tick-borne encephalitis vaccine: results from a modeling study beyond 10 years after a booster dose following different primary vaccination schedules. Hum Vaccin Immunother. 2020 Sep 1;16(9):2274-2279. doi: 10.1080/21645515.2019.1700712. Epub 2020 Jan 17. PMID: 31951780; PMCID: PMC7553683. | Excluded | Intervention |
| 6. | **Erber W, Khan F, Zavadska D, Freimane Z, Dobler G, Böhmer MM, Jodar L, Schmitt HJ. Effectiveness of TBE vaccination in southern Germany and Latvia. Vaccine. 2022 Jan 31;40(5):819-825. doi: 10.1016/j.vaccine.2021.12.028. Epub 2021 Dec 21. PMID: 34952753.** | **Included** | **-** |
| 7. | Hansson KE, Rosdahl A, Insulander M, Vene S, Lindquist L, Gredmark-Russ S, Askling HH. Tick-borne Encephalitis Vaccine Failures: A 10-year Retrospective Study Supporting the Rationale for Adding an Extra Priming Dose in Individuals Starting at Age 50 Years. Clin Infect Dis. 2020 Jan 2;70(2):245-251. doi: 10.1093/cid/ciz176. PMID: 30843030; PMCID: PMC6938976. | Excluded | Methodology |
| 8. | https://clinicaltrials.gov/ct2/show/NCT02318069 | Excluded | Lack of access |
| 9. | https://trialsearch.who.int/Trial2.aspx?TrialID=EUCTR2018-004674-94-LV | Excluded | Lack of access |
| 10. | **Kantele A, Rombo L, Vene S, Kundi M, Lindquist L, Erra EO. Three-dose versus four-dose primary schedules for tick-borne encephalitis (TBE) vaccine FSME-immun for those aged 50 years or older: A single-centre, open-label, randomized controlled trial. Vaccine. 2022 Feb 23;40(9):1299-1305. doi: 10.1016/j.vaccine.2022.01.022. Epub 2022 Jan 31. PMID: 35101266.** | **Included** | **-** |
| 11. | Kollaritsch H, Paulke-Korinek M, Holzmann H, Hombach J, Bjorvatn B, Barrett A. Vaccines and vaccination against tick-borne encephalitis. Expert Rev Vaccines. 2012 Sep;11(9):1103-19. doi: 10.1586/erv.12.86. PMID: 23151167. | Excluded | Methodology |
| 12. | **Konior R, Brzostek J, Poellabauer EM, Jiang Q, Harper L, Erber W. Seropersistence of TBE virus antibodies 10 years after first booster vaccination and response to a second booster vaccination with FSME-IMMUN 0.5mL in adults. Vaccine. 2017 Jun 16;35(28):3607-3613. doi: 10.1016/j.vaccine.2017.03.059. Epub 2017 May 22. PMID: 28545923.** | **Included** | **-** |
| 13. | Lotrič-Furlan S, Bogovič P, Avšič-Županc T, Jelovšek M, Lusa L, Strle F. Tick-borne encephalitis in patients vaccinated against this disease. J Intern Med. 2017 Aug;282(2):142-155. doi: 10.1111/joim.12625. Epub 2017 May 23. PMID: 28440879. | Excluded | Methodology |
| 14. | **Nygren TM, Pilic A, Böhmer MM, Wagner-Wiening C, Wichmann O, Harder T, Hellenbrand W. Tick-borne encephalitis vaccine effectiveness and barriers to vaccination in Germany. Sci Rep. 2022 Jul 9;12(1):11706. doi: 10.1038/s41598-022-15447-5. PMID: 35810184; PMCID: PMC9271034.** | **Included** | **-** |
| 15. | **Poellabauer E, Angermayr R, Behre U, Zhang P, Harper L, Schmitt HJ, Erber W. Seropersistence and booster response following vaccination with FSME-IMMUN in children, adolescents, and young adults. Vaccine. 2019 May 27;37(24):3241-3250. doi: 10.1016/j.vaccine.2019.03.032. Epub 2019 Mar 27. PMID: 30928173.** | **Included** | **-** |
| 16. | Prymula R, Pöllabauer EM, Pavlova BG, Löw-Baselli A, Fritsch S, Angermayr R, Geisberger A, Barrett PN, Ehrlich HJ. Antibody persistence after two vaccinations with either FSME-IMMUN® Junior or ENCEPUR® Children followed by third vaccination with FSME-IMMUN® Junior. Hum Vaccin Immunother. 2012 Jun;8(6):736-42. doi: 10.4161/hv.20058. Epub 2012 Jun 1. PMID: 22699436. | Excluded | Population |
| 17. | Santonja I, Stiasny K, Essl A, Heinz FX, Kundi M, Holzmann H. Tick-borne encephalitis in vaccinated patients: a retrospective case-control study and analysis of vaccination field effectiveness in Austria from 2000 to 2018. J Infect Dis. 2022 Mar 2:jiac075. doi: 10.1093/infdis/jiac075. Epub ahead of print. PMID: 35235953. | Excluded | Lack of access |
| 18. | Schosser R, Reichert A, Mansmann U, Unger B, Heininger U, Kaiser R. Irregular tick-borne encephalitis vaccination schedules: the effect of a single catch-up vaccination with FSME-IMMUN. A prospective non-interventional study. Vaccine. 2014 Apr 25;32(20):2375-81. doi: 10.1016/j.vaccine.2014.01.072. Epub 2014 Mar 6. PMID: 24613521. | Excluded | Intervention |
| 19. | Wanke K, Von Braun A, Haberli L, Mekker A, Steffen P, Stiasny K, Heinz F, Unger B, Karrer U. Immunogenicity and safety of tick-borne encephalitis vaccination in healthy elderly individuals. Clinical microbiology and infection, 2012, 18, 246 | Excluded | Lack of access |
| 20. | **Wittermann C, Izu A, Petri E, Gniel D, Fragapane E. Five year follow-up after primary vaccination against tick-borne encephalitis in children. Vaccine. 2015 Apr 8;33(15):1824-9. doi: 10.1016/j.vaccine.2015.02.038. Epub 2015 Feb 26. PMID: 25728316.** | **Included** | **-** |
| 21. | Zavadska D, Odzelevica Z, Karelis G, Liepina L, Litauniece ZA, Bormane A, Lucenko I, Perevoscikovs J, Bridina L, Veide L, Krumina A, Storozenko J, Erber W, Htar MTT, Schmitt HJ. Tick-borne encephalitis: A 43-year summary of epidemiological and clinical data from Latvia (1973 to 2016). PLoS One. 2018 Nov 13;13(11):e0204844. doi: 10.1371/journal.pone.0204844. PMID: 30422984; PMCID: PMC6233910. | Excluded | Methodology |
| 22. | **Zens KD, Haile SR, Schmidt AJ, Altpeter ES, Fehr JS, Lang P. Retrospective, matched case-control analysis of tickborne encephalitis vaccine effectiveness by booster interval, Switzerland 2006-2020. BMJ Open. 2022 Apr 22;12(4):e061228. doi: 10.1136/bmjopen-2022-061228. PMID: 35459683; PMCID: PMC9036433.** | **Included** | **-** |
